# Potential Effects of Panfacial Filler Injections on Facelift Surgery and Outcomes: Survey Results of the Members of the Aesthetic Society

**DOI:** 10.1093/asjof/ojad027.002

**Published:** 2023-04-14

**Authors:** Lance DeRoss, Iliana Sweis, Shreya Raman, Pravin Patel

**Affiliations:** University of Illinois at Chicago, Chicago, IL; University of Illinois at Chicago, Northbrook, IL; University of Illinois at Chicago, Chicago, IL; University of Illinois at Chicago, Chicago, IL

## Abstract

**Goals/Purpose:**

Facial soft tissue filler injections are being performed in the United States with increasing popularity. Even though there has been a trend towards noninvasive facial rejuvenation, surgery remains the standard of care for long lasting results for treatment of facial aging. Despite the well-known complications of soft tissue fillers, there remains a paucity of data regarding the potential adverse effects of prior repetitive panfacial soft tissue fillers on the technical aspects or outcome of facelift surgeries. The aim of this study was to understand the observations of the national aesthetic surgery community regarding facelift surgery in patients who have undergone repetitive panfacial fillers prior to their surgery. The primary outcome was to determine if the aesthetic community believed that a patient’s history of panfacial fillers led to potential difficulties or complications with their facelift procedure, so as to develop safer guidelines when performing surgery on these patients.

**Methods/Technique:**

The authors developed a survey to examine the experiences among members of the Aesthetic Society with patients who have a history of repetitive panfacial fillers and then undergo facelift surgery. More specifically, it was to understand if a history of repetitive panfacial fillers had potential effects on the surgery itself and the post-operative outcomes. This survey contained 10 questions in total, included closed and open-ended questions, and was sent through email to 4,180 Aesthetic Society members. The survey was e-mailed twice, first in March 2022 and again in May 2022. The data was tabulated and examined to analyze the current techniques and perceived outcomes with repetitive panfacial fillers and facelifts. Categorial variables were reported as frequencies/percentages.

**Results/Complications:**

In total, there were 156 responses, which was a 3.7% response rate. The majority of the respondents (80.8%) believed that less than 60% of their facelift patients had previous repetitive panfacial filler injections. Approximately one-half (51.9%) reported that a history of panfacial filler injections increased the difficulty of performing facelifts. A larger subset (39.7%) of respondents believed that a history of panfacial fillers increased complication rates, compared to the remaining who either disagreed (28.9%) or were unsure (31.4%). The most common complications following facelift surgery in those patients with known filler history included the undesirable palpability or visibility of filler (32.7%), compromised flap vascularity (15.4%), and decreased longevity of the lifting effect (9.6%).

**Conclusion:**

The outcome of this survey identified that there may be a potential association with repetitive panfacial filler injections and outcomes following facelift surgery, although the exact effect on postoperative outcomes remained unclear. Large prospectively designed studies are needed to capture objective data comparing facelift patients with history of repetitive panfacial fillers with those facelift patients who have never had injectables. Given these results, the authors encourage careful history-taking to elicit an accurate filler injection record including complications after filler injections, as well as a thorough discussion with these patients preoperatively regarding the potential of panfacial fillers on the facelift procedure and potential outcomes.

